# The relationship between the neural computations for speech and music perception is context-dependent: an activation likelihood estimate study

**DOI:** 10.3389/fpsyg.2015.01138

**Published:** 2015-08-11

**Authors:** Arianna N. LaCroix, Alvaro F. Diaz, Corianne Rogalsky

**Affiliations:** Communication Neuroimaging and Neuroscience Laboratory, Department of Speech and Hearing Science, Arizona State UniversityTempe, AZ, USA

**Keywords:** music perception, speech perception, fMRI, meta-analysis, Broca's area

## Abstract

The relationship between the neurobiology of speech and music has been investigated for more than a century. There remains no widespread agreement regarding how (or to what extent) music perception utilizes the neural circuitry that is engaged in speech processing, particularly at the cortical level. Prominent models such as Patel's Shared Syntactic Integration Resource Hypothesis (SSIRH) and Koelsch's neurocognitive model of music perception suggest a high degree of overlap, particularly in the frontal lobe, but also perhaps more distinct representations in the temporal lobe with hemispheric asymmetries. The present meta-analysis study used activation likelihood estimate analyses to identify the brain regions consistently activated for music as compared to speech across the functional neuroimaging (fMRI and PET) literature. Eighty music and 91 speech neuroimaging studies of healthy adult control subjects were analyzed. Peak activations reported in the music and speech studies were divided into four paradigm categories: passive listening, discrimination tasks, error/anomaly detection tasks and memory-related tasks. We then compared activation likelihood estimates within each category for music vs. speech, and each music condition with passive listening. We found that listening to music and to speech preferentially activate distinct temporo-parietal bilateral cortical networks. We also found music and speech to have shared resources in the left pars opercularis but speech-specific resources in the left pars triangularis. The extent to which music recruited speech-activated frontal resources was modulated by task. While there are certainly limitations to meta-analysis techniques particularly regarding sensitivity, this work suggests that the extent of shared resources between speech and music may be task-dependent and highlights the need to consider how task effects may be affecting conclusions regarding the neurobiology of speech and music.

## Introduction

The relationship between the neurobiology of speech and music has been investigated and debated for nearly a century. (Henschen, 1924; Luria et al., 1965; Frances et al., 1973; Peretz, 2006; Besson et al., 2011). Early evidence from case studies of brain-damaged individuals suggested a dissociation of aphasia and amusia (Yamadori et al., 1977; Basso and Capitani, 1985; Peretz et al., 1994, 1997; Steinke et al., 1997; Patel et al., 1998b; Tzortzis et al., 2000; Peretz and Hyde, 2003). However, more recent patient work examining specific aspects of speech and music processing indicate at least some overlap in deficits across the two domains. For example, patients with Broca's aphasia have both linguistic and harmonic structure deficits, and patients with amusia exhibit pitch deficits in both speech and music (Patel, 2003, 2005, 2013). Electrophysiological (e.g., ERP) studies also suggest shared resources between speech and music; for example, syntactic and harmonic violations elicit indistinguishable ERP responses such as the P600 response, which is hypothesized to originate from anterior temporal or inferior frontal regions (Patel et al., 1998a; Maillard et al., 2011; Sammler et al., 2011). Music perception also interacts with morphosyntactic representations of speech: the early right anterior negativity (ERAN) ERP component sensitive to chord irregularities interacts with the left anterior negativity's (LAN's) response to morphosyntactic violations or irregularities (Koelsch et al., 2005; Steinbeis and Koelsch, 2008b; Koelsch, 2011).

Several studies of trained musicians and individuals with absolute pitch also suggest an overlap between speech and music as there are carry-over effects of musical training onto speech processing performance (e.g., Oechslin et al., 2010; Elmer et al., 2012; for a review see Besson et al., 2011).

There is a rich literature of electrophysiological and behavioral work regarding the relationship between music and language (for reviews see Besson et al., 2011; Koelsch, 2011; Patel, 2012, 2013; Tillmann, 2012; Slevc and Okada, 2015). This work has provided numerous pieces of evidence of overlap between the neural resources of speech and music, including in the brainstem, auditory cortex and frontal cortical regions (Koelsch, 2011). This high degree of interaction between speech and music coincides with Koelsch et al.'s view that speech and music, and therefore the brain networks supporting them, cannot be separated because of their numerous shared properties, i.e., there is a “music-speech continuum” (Koelsch and Friederici, 2003; Koelsch and Siebel, 2005; Koelsch, 2011). However, evidence from brain-damaged patients suggests that music and speech abilities may dissociate, although there are also reports to the contrary (see above). Patel's (2003, 2008, 2012) Shared Syntactic Integration Resource Hypothesis (SSIRH) is in many ways a remedy to the shared-vs.-distinct debate in the realm of structural/syntactic processing. Stemming in part from the patient and electrophysiological findings, Patel proposes that language and music utilize overlapping cognitive resources but also have unique neural representations. Patel proposes that the shared resources reside in the inferior frontal lobe (i.e., Broca's area) and that distinct processes for speech and music reside in the temporal lobes (Patel, 2003).

The emergence of functional neuroimaging techniques such as fMRI have continued to fuel the debate over the contributions of shared vs. distinct neural resources for speech and music. FMRI lacks the high temporal resolution of electrophysiological methods and can introduce high levels of ambient noise potentially contaminating recorded responses to auditory stimuli. However, the greater spatial resolution of fMRI may provide additional information regarding the neural correlates of speech and music, and MRI scanner noise can be minimized using sparse sampling scanning protocols and reduced-noise continuous scanning techniques (Peelle et al., 2010). Hundreds of fMRI papers have investigated musical processes, and thousands have investigated the neural substrates of speech. Conversely, to our knowledge and as Slevc and Okada (2015) noted, only a few studies have directly compared activations to hierarchical speech and music (i.e., sentences and melodies) using fMRI (Abrams et al., 2011; Fedorenko et al., 2011; Rogalsky et al., 2011). Findings from these studies conflict with the ERP literature (e.g., Koelsch, 2005; Koelsch et al., 2005) in that the fMRI studies identify distinct neuroanatomy and/or activation response patterns for music and speech processing, although there are notable differences across these studies, particularly relating to the involvement of Broca's area in speech and music.

The differences found across neuroimaging studies regarding the overlap of the neural correlates of speech and music likely arise from the tasks used in each of these studies. For example, Rogalsky et al. used passive listening and found no activation of Broca's area to either speech or music compared to rest. Conversely, Fedorenko et al. used a reading/memory probe task for sentences and an emotional ranking for music and found Broca's area to be preferentially activated by speech but also activated by music compared to rest. There is also evidence that the P600, the ERP component that is sensitive to both speech and music violations, is only present when subjects are actively attending to the stimulus (Besson and Faita, 1995; Brattico et al., 2006; Koelsch, 2011). The inclusion of a task may affect not only the brain regions involved, but also reliability of results: an fMRI study of visual tasks reported that tasks with high attentional loads also had the highest reliability measures compared to passive conditions (Specht et al., 2003). This finding in the visual domain suggests the possibility that greater (within and between) subject variability in passive listening conditions may lead to null effects in group-averaged results.

Given the scarcity of within-subject neuroimaging studies of speech and music, it is particularly critical to examine across-study, between-subjects findings to build a better picture regarding the neurobiology of speech and music. A major barrier in interpreting between-subject neuroimaging results is the variety of paradigms and tasks used to investigate speech and music neural resources. Most scientists studying the neurobiology of speech and/or music would likely agree that they are interested in understanding the neural computations employed in naturalistic situations that are driven by the input of speech or music, and the differences between the two. However, explicit tasks such as discrimination or error detection are often used to drive brain responses in part by increasing the subject's attention to the stimuli and/or particular aspects of the stimuli. This may be problematic: the influence of task demands on the functional neuroanatomy recruited by speech is well documented (e.g., Baker et al., 1981; Noesselt et al., 2003; Scheich et al., 2007; Geiser et al., 2008; Rogalsky and Hickok, 2009) and both speech and music processing engage domain-general cognitive, memory, and motor networks in likely distinct, but overlapping ways (Besson et al., 2011). Task effects are known to alter inter and intra hemisphere activations to speech (Noesselt et al., 2003; Tervaniemi and Hugdahl, 2003; Scheich et al., 2007; Geiser et al., 2008; Rogalsky and Hickok, 2009). For example, there is evidence that right hemisphere fronto-temporal-parietal networks are significantly activated during an explicit task (rhythm judgment) with speech stimuli but not during passive listening to the same stimuli (Geiser et al., 2008). The neurobiology of speech perception, and auditory processing more generally, also can vary based on the type of explicit task even when the same stimuli are used across tasks (Platel et al., 1997; Ni et al., 2000; Von Kriegstein et al., 2003; Geiser et al., 2008; Rogalsky and Hickok, 2009). This phenomenon is also well documented in the visual domain (Corbetta et al., 1990; Chawla et al., 1999; Cant and Goodale, 2007). For example, in the speech domain, syllable discrimination and single-word comprehension performance (as measured by a word-picture matching task) doubly dissociate in stroke patients with aphasia (Baker et al., 1981). Syllable discrimination implicates left-lateralized dorsal frontal-parietal networks, while speech comprehension and passive listening tasks engage mostly mid and posterior temporal regions (Dronkers et al., 2004; Schwartz et al., 2012; Rogalsky et al., 2015). Similarly, contextual effects have been reported regarding pitch: when pitch is needed for linguistic processing, such as in a tonal language, there is a left hemisphere auditory cortex bias, while pitch processing in a melody discrimination task yields a right hemisphere bias (Zatorre and Gandour, 2008). Another example of the importance of context in pitch processing is in vowel perception: vowels and tones have similar acoustic features and when presented in isolation (i.e., just a vowel, not in a consonant-vowel (CV) pair as would typically be perceived in everyday life) no significant differences have been found in temporal lobe activations (Jäncke et al., 2002). However, there is greater superior temporal activation for CVs than tones suggesting that the context of the vowel modulates the temporal networks activated (Jäncke et al., 2002).

One way to reduce the influence of a particular paradigm or task is to use meta-analysis techniques to identify areas of activation that consistently activate to a particular stimulus (e.g., speech, music) across a range of tasks and paradigms. Besson and Schön (2001) noted that meta-analyses of neuroimaging data would provide critical insight into the relationship between the neurobiology of language and music. They also suggested that meta-analyses of music-related neuroimaging data were not feasible due to the sparse number of relevant studies. Now, almost 15 years later, there is a large enough corpus of neuroimaging work to conduct quantitative meta-analyses of music processing with sufficient power. In fact, such meta-analyses have begun to emerge, for specific aspects of musical processing, in relation to specific cognitive functions [e.g., Slevc and Okada's (2015) cognitive control meta-analysis in relation to pitch and harmonic ambiguity], in addition to extensive qualitative reviews (e.g., Tervaniemi, 2001; Jäncke, 2008; Besson et al., 2011; Grahn, 2012; Slevc, 2012; Tillmann, 2012).

The present meta-analysis addresses the following outstanding questions: (1) has functional neuroimaging identified significant distinctions between the functional neuroanatomy of speech and music and (2) how do specific types of tasks affect how music recruits speech-processing networks? We then discuss the implications of our findings for future investigations of the neural computations of language and music.

## Materials and methods

An exhaustive literature search was conducted via Google Scholar to locate published fMRI and PET studies reporting activations to musical stimuli. The following search terms were used to locate papers about music: “fMRI music,” “fMRI and music,” “fMRI pitch,” and “fMRI rhythm.” To the best of our knowledge, all relevant journal research articles have been collected for the purposes of this meta-analysis.

All journal articles that became part of the meta-analysis reported peak coordinates for relevant contrasts. Peak coordinates reported in the papers identified by the searches were divided into four categories that encompassed the vast majority of paradigms used in the articles: music passive listening, music discrimination, music error detection, and music memory^1^. Passive listening studies included papers in which participants listened to instrumental melodies or tone sequences with no explicit task as well as studies that asked participants to press a button when the stimulus concluded. Music discrimination studies included those that asked participants to compare two musical stimuli (e.g., related/unrelated, same/different). Music error detection studies included studies that instructed participants to identify a dissonant melody, unexpected note or deviant instrument. The music memory category included papers that asked participants to complete an n-back task, familiarity judgment, or rehearsal (covert or overt) of a melodic stimulus.

Only coordinates from healthy adult, non-musician, control subjects were included. In studies that included a patient group and a control group, only the control group's coordinates were included. Studies were excluded from the final activation likelihood estimate (ALE) if the data did not meet the requirements for being included in ALE calculations, including for the following reasons: coordinates not reported, only approximate anatomical location reported, stereotaxic space not reported, inappropriate contrasts (e.g., speech > music only), activations corresponding to participant's emotional reactions to music, studies of professional/trained musicians, and studies of children.

In addition to collecting the music-related coordinates via an exhaustive search, we also gathered a representative sample of fMRI and PET studies that reported coordinates for passive listening to intelligible speech compared to some type of non-speech control (e.g., tones, noise, rest, visual stimuli). Coordinates corresponding to the following tasks were also extracted: speech discrimination, speech detection, and speech memory. The purpose of these speech conditions is to act as comparison groups for the music groups. Coordinates for this purpose were extracted from six sources: five well-cited review papers, Price (2010), Zheng et al. (2010), Turkeltaub and Coslett (2010), Rogalsky et al. (2011), and Adank (2012) and the brain imaging meta-analysis database Neurosynth.org. The Price (2010), Zheng et al. (2010), Turkeltaub and Coslett (2010), Rogalsky et al. (2011), and Adank (2012) papers yielded a total of 42 studies that fit the aforementioned criteria. An additional 49 relevant papers were found using the Neurosynth.org database with the search criteria “speech perception,” “speech processing,” “speech,” and “auditory working memory.” These methods resulted in 91 studies in which control subjects passively listened to speech or completed an auditory verbal memory, speech discrimination, or speech detection task. The passive listening speech condition included studies in which participants listened to speech stimuli with no explicit task as well as studies that asked participants to press a button when the stimulus concluded. Papers were included in the speech discrimination category if they asked participants to compare two speech stimuli (e.g., a same/different task). The speech detection category contained papers that asked participants to detect semantic, intelligibility, or grammatical properties or detect phonological, semantic, or syntactic errors. Studies included in the speech memory category were papers that instructed participants to complete an n-back task or rehearsal (covert or overt) of a speech (auditory verbal) stimulus.

Analyses were conducted using the meta-analysis software GingerALE to calculate ALEs for each condition based on the coordinates collected (Eickhoff et al., 2009, 2012; Turkeltaub et al., 2012). All results are reported in Talairach space. Coordinates originally reported in MNI space were transformed to Talairach space using GingerALE's stereotaxic coordinate converter. Once all coordinates were in Talairach space, each condition was analyzed individually using the following GingerALE parameters: less conservative (larger) mask size, Turkeltaub nonadditive ALE method (Turkeltaub et al., 2012), subject-based FWHM (Eickhoff et al., 2009), corrected threshold of *p* < 0.05 using false discovery rate (FDR), and a minimum cluster volume of 200 mm^3^. We obtained subtraction contrasts between two given conditions by directly comparing activations between two conditions. To correct for multiple comparisons, each contrast's threshold was set to *p* < 0.05, whole-brain corrected following the FDR algorithm with p value permutations set at 10,000, and a minimum cluster size of 200 mm^3^ (Eickhoff et al., 2009). ALE statistical maps were rendered onto the Colin Talairach template brain using the software MRIcron (Rorden and Brett, 2000).

## Results

### Search results

The literature search yielded 80 music studies (76 fMRI studies, 4 PET studies) and 91 relevant speech papers (88 fMRI, 3 PET studies) meeting the inclusion criteria described above. Table 1 indicates the number of studies, subjects, and coordinates in each of the four music conditions, as well as for each of the four speech conditions.

**Table 1:** **Activations included in the present meta-analysis**.

**Condition**	**Number of studies**	**Number of subjects**	**Number of coordinates**
Music passive listening	41	540	526
Music discrimination	12	211	168
Music error detection	25	355	489
Music memory	14	190	207
Speech passive listening	31	454	337
Speech discrimination	31	405	318
Speech detection	17	317	248
Speech memory	19	259	324

### Passive listening to music vs. passive listening to speech

**Figure 1:**
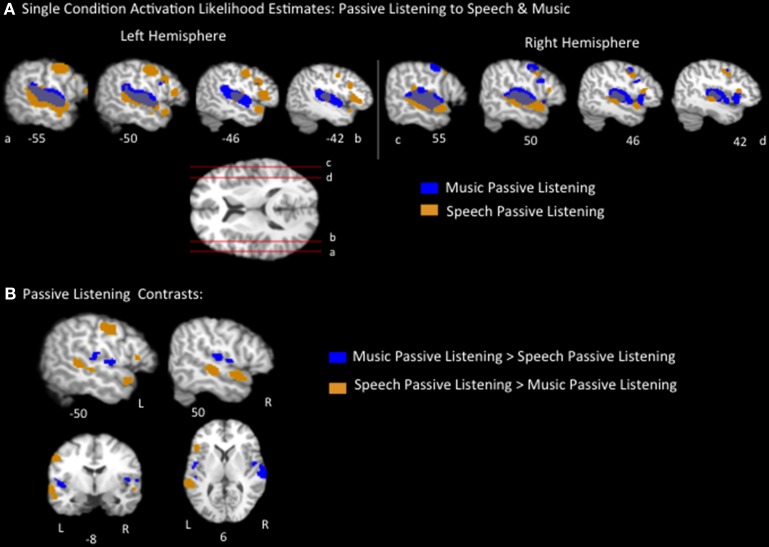
**(A)** Representative sagittal slices of the ALE for passive listening to speech, *p* < 0.05, corrected, overlaid on top of the passive music listening ALE. **(B)** Speech vs. music passive listening contrasts results, *p* < 0.05 corrected.

The music passive listening ALE identified large swaths of voxels bilaterally, spanning the length of the superior temporal gyri (STG), as well as additional smaller clusters, including in the bilateral inferior frontal gyrus (pars opercularis), bilateral postcentral gyrus, bilateral insula, left inferior parietal lobule, left medial frontal gyrus, right precentral gyrus, and right middle frontal gyrus (Figure 1A, Table 2). The speech passive listening ALE also identified bilateral superior temporal regions as well as bilateral precentral and inferior frontal (pars opercularis) regions. Notably, the speech ALE identified bilateral anterior STG, bilateral superior temporal sulcus (i.e., both banks, the middle and superior temporal gyri) and left inferior frontal gyrus (pars triangularis) regions not identified by the music ALE (Figure 1A, Table 2). ALEs used a threshold of *p* < 0.05, FDR corrected.

**Table 2:** **Locations, peaks and cluster size for significant voxel clusters for each condition's ALE and for each contrast of interest**.

**Condition**	**Anatomical locations**	**Peak coordinates**	**Voxels**
Music passive listening	Left inferior frontal gyrus (pars opercularis)^*^	−46, 10, 26	32
	Left medial frontal gyrus^*^, left subcallosal gyrus	−2, 26,−14	65
	Left medial frontal gyrus^*^	−2, 2, 62	48
	Left postcentral gyrus^*^, left inferior parietal lobule	−34,−36, 54	27
	Left superior temporal gyrus^*^, left transverse temporal gyrus, left middle temporal gyrus, left insula	−52,−20, 6	2073
	Right inferior frontal gyrus^*^	48, 10, 28	43
	Right precentral gyrus^*^, right postcentral gyrus, right middle frontal gyrus	52,−2, 44	173
	Right superior temporal gyrus^*^, right transverse temporal gyrus, right middle temporal gyrus, right insula	58,−20, 6	2154
	Right insula^*^, right inferior frontal gyrus, right precentral gyrus	42, 14, 0	206
	Right lingual gyrus^*^, right culmen	16,−54,−2	27
Music discrimination	Left medial frontal gyrus^*^, left middle frontal gyrus	−8,−4, 58	224
	Left precentral gyrus^*^, left postcentral gyrus, left inferior parietal lobule	−48,−12, 48	259
	Left precentral gyrus^*^, left inferior frontal gyrus (pars opercularis)	−50, 2, 26	67
	Left superior temporal gyrus^*^, left transverse temporal gyrus, left precentral gyrus	−54,−16, 8	239
	Left superior temporal gyrus^*^, left middle temporal gyrus	−58,−34, 8	92
	Left insula^*^, left inferior frontal gyrus (pars triangularis)	−34, 22, 2	48
	Left cerebellum^*^	−28,−62,−24	127
	Right inferior frontal gyrus^*^, right middle frontal gyrus	52, 12, 28	58
	Right precentral gyrus^*^, right middle frontal gyrus	46,−6, 44	170
	Right superior temporal gyrus^*^, right middle temporal gyrus	62,−24, 8	310
	Right superior temporal gyrus^*^, right precentral gyrus, right insula	50, 6,−2	91
Music error detection	Left medial frontal gyrus^*^	−4,−4, 58	49
	Left superior temporal gyrus^*^, Let transverse temporal gyrus,Left postcentral gyrus, left insula	−50,−18, 8	1448
	Left inferior parietal lobule^*^, left supramarginal gyrus, left angular gyrus	−40,−48, 40	41
	Left lentiform nucleus^*^, left putamen	−22, 6, 10	263
	Right middle frontal gyrus^*^	36, 42, 18	43
	Right middle frontal gyrus^*^, right precentral gyrus	32,−4, 56	35
	Right superior frontal gyrus^*^, right medial frontal gyrus, left superior frontal gyrus, left medial frontal gyrus	2, 10, 52	95
	Right superior temporal gyrus^*^, right transverse temporal gyrus, right insula, right precentral gyrus, right middle temporal gyrus, right claustrum	50,−18, 6	1228
	Right parahippocampal gyrus^*^	22,−14,−12	36
	Right inferior parietal lobule^*^, right supramarginal gyrus	36,−44, 40	103
	Right insula^*^, right inferior frontal gyrus	32, 22, 12	329
	Right lentiform nucleus^*^, right putamen, right caudate	18, 6, 12	144
	Right thalamus^*^	12,−16, 8	33
	Right cerebellum^*^	26,−50,−26	28
Music memory	Left inferior frontal gyrus (pars opercularis)^*^, left precentral gyrus, left middle frontal gyrus	−50, 4, 26	206
	Left inferior frontal gyrus (pars triangularis^*^, pars orbitalis), left insula	−34, 24,−2	57
	Left inferior frontal gyrus (pars triangularis)^*^	−44, 26, 10	25
	Left medial frontal gyrus^*^	−4, 52, 12	31
	Left middle frontal gyrus^*^	−32, 4, 54	29
	Left precentral gyrus^*^	−44,−10, 42	33
	Left superior frontal gyrus^*^, left medial frontal gyrus, right superior frontal gyrus, right medial frontal gyrus	−0, 12, 50	373
	Left middle temporal gyrus^*^	−50,−20,−10	72
	Left middle temporal gyrus^*^, left superior temporal gyrus	−46, 4,−18	35
	Left inferior parietal lobule^*^, left superior temporal gyrus, left middle temporal gyrus, left supramarginal gyrus	−48,−44, 22	224
	Left thalamus^*^	−14,−14, 14	37
	Right inferior frontal gyrus^*^, right insula, right claustrum	32, 26, 8	90
	Right middle frontal gyrus^*^	38, 44, 14	27
	Right superior temporal gyrus^*^, right middle temporal gyrus	54,−38, 10	35
	Right parahippocampal gyrus^*^, right hippocampus	30,−10,−20	35
	Right cerebellum^*^	30,−56,−18	47
Speech passive listening	Left inferior frontal gyrus (pars triangularis, pars opercularis)^*^, left insula, left precentral gyrus	−44, 20, 8	296
	Left inferior frontal gyrus (pars triangularis^*^, pars opercularis), left middle frontal gyrus, left precentral gyrus	−48, 10, 28	162
	Left precentral gyrus^*^, left postcentral gyrus	−52,−10, 40	294
	Left medial frontal gyrus^*^, left superior frontal gyrus, left medial frontal gyrus, left cingulate gyrus	−8, 8, 50	164
	Left superior temporal gyrus^*^, Left middle temporal gyrus, left postcentral gyrus, left transverse temporal gyrus	−58,−14,−2	2101
	Left superior temporal gyrus^*^	−46, 12,−14	107
	Left fusiform gyrus^*^, left inferior occipital gyrus, left middle occipital gyrus	−38,−78,−12	35
	Right inferior frontal gyrus^*^, right insula, right precentral gyrus	44, 18, 10	81
	Right middle frontal gyrus^*^, right precentral gyrus, right inferior frontal gyrus	46, 2, 38	118
	Right superior temporal gyrus^*^, right middle temporal gyrus, Right insula, right precentral gyrus, right transverse temporal gyrus	−52, 20, 0	1800
Speech discrimination	Left inferior frontal gyrus (pars orbitalis^*^, pars triangularis), left insula, left middle frontal gyrus	−38, 26,−4	115
	Left inferior frontal gyrus (pars triangularis^*^, pars opercularis), left precentral gyrus	−44, 20, 10	44
	Left middle frontal gyrus^*^, left inferior frontal gyrus (pars triangularis, pars opercularis)	−46, 16, 30	187
	Left middle frontal gyrus^*^, left precentral gyrus	−46,−0, 42	26
	Left superior temporal gyrus^*^, left postcentral gyrus, left transverse temporal gyrus, left middle temporal gyrus	−58,−20, 4	1737
	Left thalamus^*^, left caudate	−14,−16, 10	147
	Left cerebellum	−38,−60,−16	36
	Right inferior frontal gyrus^*^, right precentral gyrus, right insula	46, 20, 4	38
	Right superior temporal gyrus^*^, right middle temporal gyrus, right transverse temporal gyrus, right insula	58,−14, 0	1223
	Right precuneus^*^, right cuneus	4,−78, 38	34
Speech detection	Left inferior frontal gyrus (pars opercularis)^*^, left middle frontal gyrus, left insula	−48, 10, 22	361
	Left inferior frontal gyrus (pars triangularis)^*^	−48, 28, 12	101
	Left inferior frontal gyrus (pars triangularis^*^, pars orbitalis), left insula	−34, 24, 2	61
	Left postcentral gyrus^*^, left precentral gyrus	−50,−12, 46	92
	Left medial frontal gyrus^*^, left superior frontal gyrus	−6,−6, 60	54
	Left superior temporal gyrus^*^, left middle temporal gyrus, left transverse temporal gyrus	−60,−22,−2	1010
	Left superior temporal gyrus^*^, left supramarginal gyrus, left inferior parietal lobule	−60,−42, 20	66
	Left superior temporal gyrus^*^, left middle temporal gyrus	−50, 12,−14	34
	Left superior temporal gyrus^*^	−42, 18,−24	28
	Left transverse temporal gyrus^*^, left superior temporal gyrus	−36,−30, 12	38
	Left precuneus^*^, left superior parietal lobule, left inferior parietal lobule	−30,−62, 40	66
	Right inferior frontal gyrus^*^, right insula	34, 24, 6	62
	Right inferior frontal gyrus^*^	40, 24,−4	31
	Right inferior frontal gyrus^*^	52, 8, 22	29
	Right superior temporal gyrus^*^, right transverse temporal gyrus, right middle temporal gyrus	58,−14, 4	788
	Right middle frontal gyrus^*^	48, 14, 32	36
Speech memory	Left middle frontal gyrus^*^, left inferior frontal gyrus (pars triangularis, pars opercularis), left precentral gyrus	−50, 22, 22	476
	Left superior frontal gyrus^*^, left medial frontal gyrus, right medial frontal gyrus, right superior frontal gyrus	−2, 4, 56	73
	Left precentral gyrus^*^, left postcentral gyrus	−50,−10, 44	127
	Left insula^*^, left inferior frontal gyrus (pars triangularis), left claustrum	−30, 18, 4	39
	Left superior temporal gyrus^*^, left middle temporal gyrus, left insula	−62,−24, 6	937
	Left superior temporal gyrus^*^, left middle temporal gyrus	−50, 10,−10	62
	Left superior parietal lobule^*^, left precuneus, left inferior parietal lobule	−30,−62, 48	109
	Left inferior parietal lobule^*^	−40,−46, 50	93
	Left caudate^*^, left thalamus	−16,−2, 16	36
	Left cerebellum^*^, left fusiform gyrus	−40,−44,−20	67
	Right superior temporal gyrus^*^, right middle temporal gyrus, right transverse temporal gyrus	58,−14, 0	773
	Right superior temporal gyrus^*^, right middle temporal gyrus	48, 8,−14	58
	Right cerebellum	24,−64,−16	50
Music passive > speech passive	Left insula^*^, left superior temporal gyrus	−44,−6, 2	148
	Left superior temporal gyrus^*^, left insula, left middle temporal gyrus	−42,−40, 14	146
	Left subcallosal gyrus^*^, left medial frontal gyrus, left anterior cingulate	−4, 22,−14	53
	Right inferior frontal gyrus^*^, right insula	44, 18,−2	49
	Right superior temporal gyrus^*^, right postcentral gyrus, right transverse temporal gyrus, right precentral gyrus, right insula	66,−20, 10	457
Music passive < speech passive	Left inferior frontal gyrus (pars triangularis)^*^, left precentral gyrus	−42, 30, 2	177
	Left precentral gyrus^*^, left postcentral gyrus	−56,−10, 40	191
	Left middle temporal gyrus^*^, left inferior temporal gyrus, left superior temporal gyrus	−56,−12,−12	856
	Left middle temporal gyrus^*^, left superior temporal gyrus	−50, 6,−18	91
	Left cingulate gyrus^*^, left medial frontal gyrus, left superior frontal gyrus	−10, 4, 46	70
	Right middle temporal gyrus^*^, right superior temporal gyrus, right insula	56,−22,−8	277
	Right middle temporal gyrus^*^, right superior temporal gyrus	52, 2,−12	167
Music discrimination > speech discrimination	Left inferior frontal gyrus (pars opercularis)^*^, left precentral gyrus	−52, 4, 24	56
	Left postcentral gyrus^*^, left inferior parietal lobule, left precentral gyrus	−48,−18, 44	253
	Left medial frontal gyrus^*^, left superior frontal gyrus, right medial frontal gyrus, right superior frontal gyrus	−8,−6, 54	224
	Left superior temporal gyrus^*^, left transverse temporal gyrus, left precentral gyrus	−52,−10, 8	122
	Left cerebellum	−28,−64,−28	114
	Right inferior frontal gyrus^*^, right middle frontal gyrus	50, 8, 26	53
	Right precentral gyrus^*^, right middle frontal gyrus	36,−6, 42	170
	Right precentral gyrus^*^, right insula, right superior temporal gyrus	48, 4, 8	91
	Right superior temporal gyrus^*^, right transverse temporal gyrus	66,−26, 10	93
Music discrimination < speech discrimination	Left middle temporal gyrus^*^, left superior temporal gyrus	−62,−18,−8	456
	Right middle temporal gyrus^*^, right superior temporal gyrus	66,−8,−4	38
Music detection > speech detection	Left insula^*^, left superior temporal gyrus, left precentral gyrus	−40,−16, 8	126
	Left insula^*^, left superior temporal gyrus	−42, 4,−6	76
	Left superior temporal gyrus^*^, left transverse temporal gyrus	−48,−34, 16	131
	Right insula^*^, right transverse temporal gyrus, right superior temporal gyrus	44,−10,−4	507
	Right middle frontal gyrus^*^, right insula	38, 16, 24	78
	Right middle frontal gyrus^*^, right precentral gyrus	32,−4, 54	35
Music detection < speech detection	Left inferior frontal gyrus (par opercularis)^*^	−56, 16, 20	240
	Left inferior frontal gyrus (pars triangularis)^*^	−52, 28, 12	101
	Left middle temporal gyrus^*^, left superior temporal gyrus, left transverse temporal gyrus	−60,−32,−2	561
	Left superior temporal gyrus^*^	−44, 18,−24	28
	Right middle temporal gyrus^*^, right superior temporal gyrus	62,−12,−4	361
Music memory > speech memory	Left cingulate gyrus^*^, left superior frontal gyrus, left medial frontal gyrus, right cingulate gyrus	−6, 20, 32	161
	Left superior temporal gyrus^*^, left supramarginal gyrus, left inferior parietal lobule	−46,−48, 14	45
Music memory < speech memory	Left inferior frontal gyrus (pars triangularis^*^, pars opercularis)	−54, 24, 20	80
	Left superior temporal gyrus^*^, left middle temporal gyrus	−60,−16, 6	606
	Right superior temporal gyrus^*^, right middle temporal gyrus, right transverse temporal gyrus	52,−26, 2	506
Music passive listening > music discrimination	Left insula^*^, left superior temporal gyrus	−42,−12,−4	116
	Left superior temporal gyrus^*^, left insula	−42,−42, 12	261
	Right superior temporal gyrus^*^, right insula	52,−12, 4	157
Music passive listening < music discrimination	Left medial frontal gyrus^*^, right medial frontal gyrus	−8,−6, 54	165
	Left precentral gyrus^*^, left superior temporal gyrus	−52, 2, 8	80
	Left postcentral gyrus^*^, left inferior parietal lobule, left precentral gyrus	−46,−18, 46	228
	Left cerebellum^*^	−24,−62,−24	90
	Right precentral gyrus^*^, right middle frontal gyrus	44,−6, 42	105
	Right precentral gyrus^*^, right insula, right superior temporal gyrus	50, 6, 6	30
Music passive > music error detection	Left middle temporal gyrus^*^	−58,−32, 0	82
	Left superior temporal gyrus^*^	−58,−10, 4	81
	Right precentral gyrus^*^, right middle frontal gyrus	50, 2, 48	64
	Right postcentral gyrus^*^, right superior temporal gyrus	62,−24, 16	44
	Right superior temporal gyrus^*^, right transverse temporal gyrus, right middle temporal gyrus, right precentral gyrus	60,−16, 0	336
Music passive < music error detection	Left medial frontal gyrus^*^	−4,−8, 56	30
	Left superior frontal gyrus^*^, left medial frontal gyrus, right superior frontal gyrus	−0, 8, 48	93
	Left postcentral gyrus^*^, left transverse temporal gyrus, left precentral gyrus	−52,−22, 16	79
	Left inferior parietal lobule^*^, left supramarginal gyrus	−40,−48, 38	37
	Left superior temporal gyrus^*^, left precentral gyrus	−52, 2, 4	92
	Left insula^*^, left superior temporal gyrus, left transverse temporal gyrus	−40,−28, 14	67
	Left lentiform nucleus^*^, left caudate	−18, 10, 8	211
	Right inferior parietal lobule^*^, right supramarginal gyrus	36,−50, 42	101
	Right insula^*^, right inferior frontal gyrus, right middle frontal gyrus	38, 18, 16	227
	Right insula^*^, right superior temporal gyrus	40,−20, 16	139
	Right insula^*^, right superior temporal gyrus	42,−8, 0	42
	Right caudate^*^, right lentiform nucleus	14, 6, 14	143
	Right thalamus^*^	14,−18, 6	32
	Right cerebellum^*^	28,−54,−26	28
Music passive listening > music memory	Left superior temporal gyrus^*^, left middle temporal gyrus, left insula	−54,−22, 6	943
	Right superior temporal gyrus^*^, right insula, right postcentral gyrus, right precentral gyrus right transverse temporal gyrus, right middle temporal gyrus	52,−20, 4	1350
	Right insula^*^, right inferior frontal gyrus, right superior temporal gyrus	46, 10, 2	32
Music passive listening < music memory	Left inferior frontal gyrus (pars opercularis)^*^, left precentral gyrus	−44, 4, 30	79
	Left inferior frontal gyrus (pars orbitalis)^*^, left insula	−32, 24,−6	53
	Left middle frontal gyrus^*^, left inferior frontal gyrus (pars triangularis)	−42, 18, 28	29
	Left middle frontal gyrus^*^	−32, 6, 54	29
	Left superior frontal gyrus^*^, left medial frontal gyrus, right medial frontal gyrus, right superior frontal gyrus	−0, 8, 48	329
	Left superior temporal gyrus^*^, left middle temporal gyrus	−44,−20,−10	69
	Left inferior parietal lobule^*^, left supramarginal gyrus, left superior temporal gyrus	−54,−44, 28	89
	Left thalamus^*^	−10,−16, 14	37
	Right inferior frontal gyrus^*^, right insula, right claustrum	32, 26, 4	83
	Right parahippocampal gyrus^*^, right hippocampus	32,−12,−24	35
Speech passive listening > music discrimination	Left middle temporal gyrus^*^, left superior temporal gyrus	−56,−20,−8	298
	Right middle temporal gyrus^*^, right superior temporal gyrus	56,−18,−4	308
Speech passive listening < music discrimination	Left precentral gyrus^*^, left superior temporal gyrus	−52, 2, 8	105
	Left postcentral gyrus^*^, left precentral gyrus, left inferior parietal lobule	−50,−14, 52	199
	Left cerebellum	−28,−64,−28	127
	Right inferior frontal gyrus^*^, right middle frontal gyrus	50, 10, 30	50
	Right medial frontal gyrus^*^, right superior frontal gyrus, left medial frontal gyrus, left superior frontal gyrus	2,−6, 62	166
	Right precentral gyrus^*^, right middle frontal gyrus	38,−8, 42	67
	Right superior temporal gyrus^*^	64,−26, 8	76
	Right superior temporal gyrus^*^, right precentral gyrus, right superior temporal gyrus	50, 6, 4	47
Speech passive listening > music detection	Left inferior frontal gyrus (pars triangularis^*^, pars opercularis),	−50, 22, 10	107
	Left middle frontal gyrus^*^, left precentral gyrus, left postcentral gyrus,	−54, 2, 40	138
	Left middle temporal gyrus^*^, left superior temporal gyrus, left inferior temporal gyrus	−60,−8,−10	1052
	Left superior temporal gyrus^*^	−48, 16,−16	29
	Right middle temporal gyrus^*^, right superior temporal gyrus	60,−18,−8	651
Speech passive listening < music detection	Left precentral gyrus^*^, left superior temporal gyrus	−52, 2, 6	54
	Left insula^*^, left superior temporal gyrus	−50,−20, 16	430
	Left insula^*^, left superior temporal gyrus	−40, 6,−4	31
	Left inferior parietal lobule^*^, left supramarginal gyrus	−42,−50, 38	40
	Left lentiform nucleus^*^, left claustrum, left insula	−20, 6, 6	203
	Right middle frontal gyrus^*^, right inferior frontal gyrus, right insula	42, 16, 32	220
	Right middle frontal gyrus^*^, right precentral gyrus	30,−6, 56	35
	Right superior frontal gyrus^*^, right middle frontal gyrus	32, 44, 16	40
	Right superior frontal gyrus^*^	4, 12, 54	36
	Right insula^*^, right transverse temporal gyrus, right superior temporal gyrus, right precentral gyrus	44,−12, 12	519
	Right inferior parietal lobule^*^, right supramarginal gyrus	40,−48, 38	103
	Right thalamus^*^, right caudate	8,−2, 10	142
	Right thalamus^*^	10,−14, 6	33
Speech passive listening > music memory	Left middle temporal gyrus^*^, left middle temporal gyrus, left transverse temporal gyrus	−58,−38,−4	1256
[-10pt]	Right superior temporal gyrus^*^, right transverse temporal gyrus, right middle temporal gyrus, right postcentral gyrus	58,−2, 2	1056
Speech passive listening < music memory	Left inferior frontal gyrus (pars orbitalis^*^, pars triangularis)	−32, 24,−6	31
[-10pt]	Left medial frontal gyrus^*^, left superior frontal gyrus, left cingulate, right superior frontal gyrus, right medial frontal gyrus, right cingulate	−0, 22, 46	336
	Left precentral gyrus^*^, left inferior frontal gyrus (pars opercularis)	−52, 2, 30	34
	Left precentral gyrus^*^	−38,−10, 38	32
	Left supramarginal gyrus^*^, left inferior parietal lobule, left superior temporal gyrus	−46,−46, 26	113
	Left inferior parietal lobule^*^, left postcentral gyrus	−48,−36, 48	71
	Left middle temporal gyrus^*^, left superior temporal gyrus	−46,−24,−10	44
	Right insula^*^, right inferior frontal gyrus, right claustrum	34, 22, 4	48

The x, y, z coordinates are in Talairach space and refer to the peak voxel activated in each contrast. All contrasts are thresholded at p = 0.05. Asterisks indicate anatomical location of peak voxel.

Pairwise contrasts of passive listening to music vs. passive listening to speech were calculated to identify any brain regions that were significantly activated more by speech or music, respectively. Results were as follows (*p* < 0.05, FDR corrected): the speech > music contrast identified significant regions on both banks of the bilateral superior temporal sulcus extending the length of the left temporal lobe and mid/anterior right temporal lobe, left inferior frontal lobe (pars triangularis), left precentral gyrus, and left postcentral gyrus regions. Music > speech identified bilateral insula and bilateral superior temporal/parietal operculum clusters as well as a right inferior frontal gyrus region (Figure 1B, Table 2). These results coincide with previous reports of listening to speech activating a lateral temporal network particularly in the superior temporal sulcus and extending into the anterior temporal lobe, while listening to music activated a more dorsal medial temporal-parietal network (Jäncke et al., 2002; Rogalsky et al., 2011). These results also coincide with Fedorenko et al.'s (2011) finding that Broca's area, the pars triangularis in particular, is preferentially responsive to language stimuli.

### Music tasks vs. speech tasks

**Figure 2:**
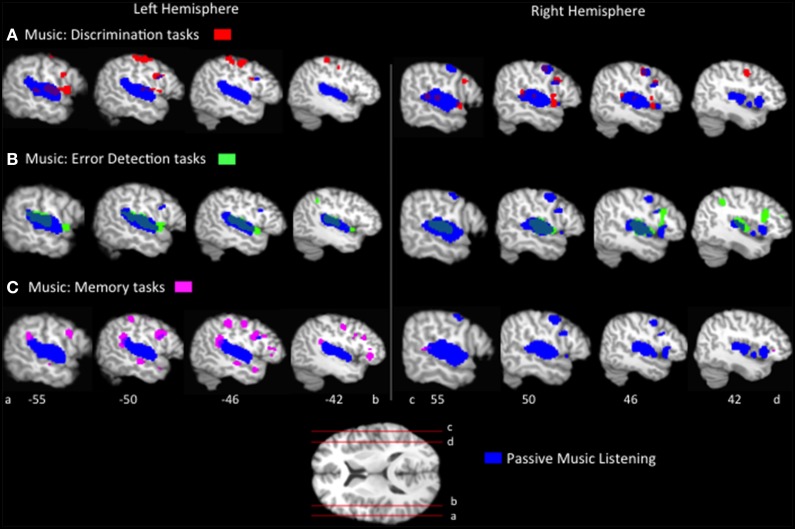
**Representative sagittal slices of the ALEs for the (A) music discrimination, (B) music error detection and (C) music memory task conditions, ***p*** < 0.05, corrected, overlaid on top of the passive music listening ALE for comparison**.

The passive listening ALE results identify distinct and overlapping regions of speech and music processing. We now turn to the question of how do these distinctions change as a function of the type of task employed? First, ALEs were computed for each music task condition, *p* < 0.05 FDR corrected (Figure 1, Table 2). The music task conditions' ALEs all significantly identified bilateral STG and bilateral precentral gyrus, and inferior parietal regions, overlapping with the passive listening music ALE (Figure 2). The tasks also activated additional inferior frontal and inferior parietal regions not identified by the passive listening music ALE; these differences are discussed in a subsequent section.

**Figure 3:**
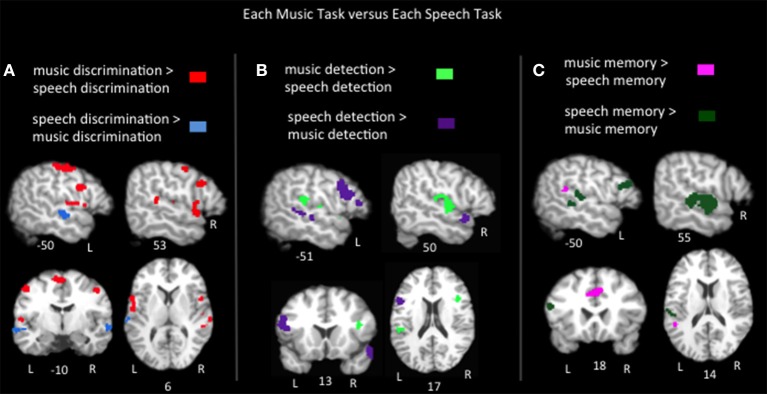
**Representative slices of the contrast results for the comparison of (A) music discrimination, (B) music error detection, and (C) music memory task conditions, compared to the corresponding speech task, ***p*** < 0.05, corrected**.

To compare the brain regions activated by each music task to those activated by speech in similar tasks, pairwise contrasts of the ALEs for each music task vs. its corresponding speech task group were calculated (Figure 3, Table 2). Music discrimination > speech discrimination identified regions including bilateral inferior frontal gyri (pars opercularis), bilateral pre and postcentral gyri, bilateral medial frontal gyri, left inferior parietal lobule, and left cerebellum, whereas speech discrimination > music discrimination identified bilateral regions in the anterior superior temporal sulci (including both superior and middle temporal gyri). Music detection > speech detection identified a bilateral group of clusters spanning the superior temporal gyri, bilateral precentral gyri, bilateral insula and bilateral inferior parietal regions, as well as clusters in the right middle frontal gyrus. Speech detection > music detection identified bilateral superior temporal sulci regions as well as left inferior frontal regions (pars triangularis and pars opercularis). Music memory > speech memory identified a left posterior superior temporal/inferior parietal region and bilateral medial frontal regions; speech memory > music memory identified left inferior frontal gyrus (pars opercularis and pars triangularis) and bilateral superior and middle temporal gyri.

In sum, the task pairwise contrasts in many ways mirror the passive listening contrast: music tasks activated more dorsal/medial superior temporal and inferior parietal regions, while speech tasks activated superior temporal sulcus regions, particularly in the anterior temporal lobe. In addition, notable differences were found in Broca's area and its right hemisphere homolog: in discrimination tasks music significantly activated Broca's area (specifically the pars opercularis) more than speech. However, in detection and memory tasks speech activated Broca's area (pars opercularis and pars triangularis) more than music. The right inferior frontal gyrus responded equally to speech and music in both detection and memory tasks, but responded more to music than speech in discrimination tasks. Also notably, in the memory tasks, music activated a lateral superior temporal/inferior parietal cluster (in the vicinity of Hickok and Poeppel's “area Spt”) more than speech while an inferior frontal cluster including the pars opercularis was activated more for speech than music. Both area Spt and the pars opercularis previously have been implicated in a variety of auditory working memory tasks (including speech and pitch working memory) in both lesion patients and control subjects (Koelsch and Siebel, 2005; Koelsch et al., 2009; Buchsbaum et al., 2011) and are considered to be part of an auditory sensory-motor integration network (Hickok et al., 2003; Hickok and Poeppel, 2004, 2007).

### Music tasks vs. passive listening to speech

**Figure 4:**
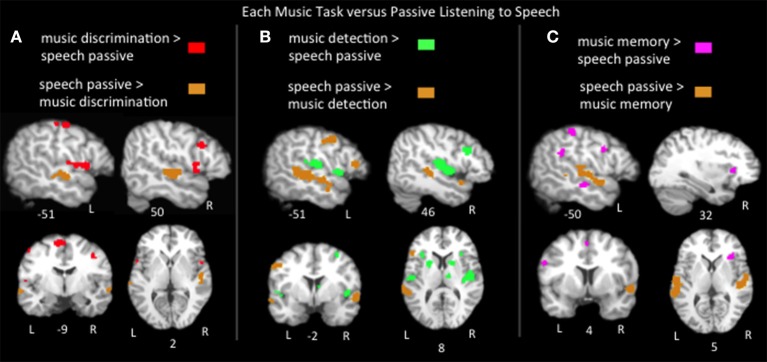
**Representative slices of the contrast results for the comparison of (A) music discrimination, (B) music error detection, (C) music memory task conditions, compared to passive listening to speech, ***p*** < 0.05, corrected**.

Findings from various music paradigms and tasks are often reported as engaging language networks because of location; a music paradigm activating Broca's area or superior temporal regions is frequently described as recruiting classic language areas. However, it is not clear if these music paradigms are in fact engaging the language networks engaged in the natural, everyday process of listening to speech. Thus, pairwise contrasts of the ALEs for listening to speech vs. the music tasks were calculated (Figure 4; Table 2). Music discrimination > speech passive listening identified regions in bilateral precentral gyri, bilateral medial frontal gyri, left postcentral gyrus, left inferior parietal lobule, left cerebellum, right inferior and middle frontal gyri, and right superior temporal gyrus. Music error detection > speech identified bilateral precentral gyri, bilateral superior temporal gyri, bilateral insula, bilateral basal ganglia, left postcentral gyrus, left cerebellum, bilateral inferior parietal lobe, right middle frontal gyrus, right inferior frontal gyrus and the right thalamus. Music memory > speech identified portions of bilateral inferior frontal gyri, bilateral medial frontal gyri, left inferior parietal lobe, left pre and postcentral gyri, and right insula. Compared to all three music tasks, speech significantly activated bilateral superior temporal sulcus regions and only activated Broca's area (specifically the pars triangularis) more than music detection. The recruitment of Broca's area and adjacent regions for music was task dependent: compared to listening to speech, music detection and discrimination activated additional bilateral inferior precentral gyrus regions immediately adjacent to Broca's area and music memory activated the left inferior frontal gyrus more than speech (in all three subregions: pars opercularis, pars triangularis, and pars orbitalis). In the right hemisphere homolog of Broca's area, all three music tasks activated this region more than listening to speech as well as adjacent regions in the right middle frontal gyrus. All together these results suggest that the recruitment of neural resources used in speech for music processing depends on the experimental paradigm. The finding of music memory tasks eliciting widespread activation in Broca's area compared to listening to speech is likely due to the inferior frontal gyrus, and the pars opercularis in particular being consistently implicated in articulatory rehearsal and working memory (Hickok et al., 2003; Buchsbaum et al., 2011, 2005), resources that are likely recruited by the music memory tasks.

### Music tasks vs. passive listening to music

**Figure 5:**
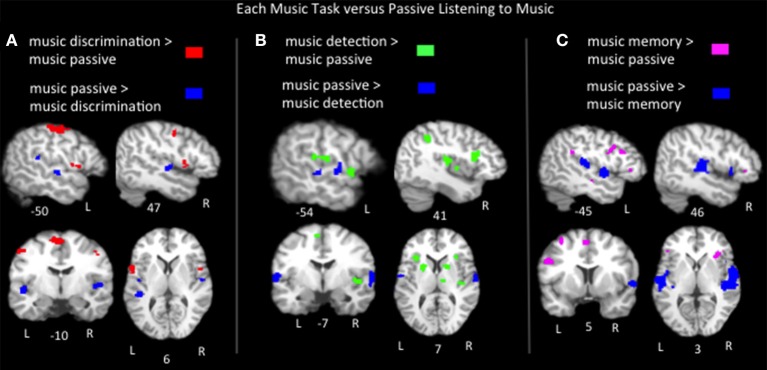
**Representative slices of the contrast results for the comparison of (A) music discrimination, (B) music error detection, (C) music memory task conditions, compared to passive listening to music, ***p*** < 0.05, corrected**.

Lastly we compared the music task ALEs to the music passive listening ALE using pairwise contrasts to better characterize task-specific activations to music. Results (*p* < 0.05, FDR corrected) include: (1) music discrimination > music listening identified bilateral inferior precentral gyri, bilateral medial frontal regions, left postcentral gyrus, left inferior parietal lobule, left cerebellum, right middle frontal gyrus and right insula (2) music error detection > music listening identified bilateral medial frontal, bilateral insula, bilateral inferior parietal areas, bilateral superior temporal gyri, bilateral basal ganglia, left pre and post central gyri, right inferior and middle frontal gyri and right cerebellum; (3) music memory > passive listening identified bilateral inferior frontal gyri (pars opercularis, triangularis and orbitalis in the left hemisphere, only the latter two in the right hemisphere), bilateral medial frontal gyri, bilateral insula, bilateral cerebellum, left middle frontal gyrus, left inferior parietal lobe, left superior and middle temporal gyri, right basal ganglia, right hippocampus and right parahippocampal gyrus (Figure 5, Table 2). The medial frontal and inferior parietal activations identified in the tasks compared to listening likely reflect increased vigilance and attention due to the presence of a task, as activation in these regions is known to increase as a function of effort and performance on tasks across a variety of stimuli types and domains (Petersen and Posner, 2012; Vaden et al., 2013). To summarize the findings in Broca's area and its right hemisphere homolog, music memory tasks activated Broca's area more than just listening to music, while music discrimination and detection tasks activated right inferior frontal gyrus regions more than listening to music. Also note that all three music tasks compared to listening to music implicate regions on the anterior bank of the inferior portion of the precentral gyrus immediately adjacent to Broca's area. Significant clusters more active for music passive listening than for each of the three task conditions are found in the bilateral superior temporal gyri (Table 2).

## Discussion

The present meta-analysis examined data from 80 functional neuroimaging studies of music and 91 studies of speech to characterize the relationship between the brain networks activated by listening to speech vs. listening to music. We also compared the brain regions implicated in three frequently used music paradigms (error detection, discrimination, and memory) to the regions implicated in similar speech paradigms to determine how task effects may change how the neurobiology of music processing is related to that of speech. We replicated across a large collection of studies' previous within-subject findings that speech activates a predominately lateral temporal network, while music preferentially activates a more dorsal medial temporal network extending into the inferior parietal lobe. In Broca's area, we found overlapping resources for passive listening to speech and music in the pars opercularis, but speech “specific” resources in pars triangularis; the right hemisphere homolog of Broca's area was equally responsive to listening to speech and music. The use of a paradigm containing an explicit task (error detection, discrimination or memory) altered the relationship between the brain networks engaged in music and speech. For example, speech discrimination tasks do not activate the pars triangularis (i.e., the region identified as “speech specific” by the passive listening contrast) more than music discrimination tasks, and both speech detection and memory tasks activate the pars opercularis (i.e., the region responding equally to music and speech passive listening) more than the corresponding music tasks, while music discrimination activates pars opercularis more than speech discrimination. These findings suggest that inferior frontal contributions to music processing, and their overlap with speech resources, may be modulated by task. The following sections discuss these findings in relation to neuroanatomical models of speech and music.

### Hemispheric differences for speech and music

The lateralization of speech and music processing has been investigated for decades. While functional neuroimaging studies report bilateral activation for both speech and music (Jäncke et al., 2002; Abrams et al., 2011; Fedorenko et al., 2011; Rogalsky et al., 2011), evidence from amusia, aphasia and other patient populations have traditionally identified the right hemisphere as critical for music and the left for basic language processes in most individuals (Gazzaniga, 1983; Peretz et al., 2003; Damasio et al., 2004; Hyde et al., 2006). Further evidence for hemispheric differences comes from asymmetries in early auditory cortex: left hemisphere auditory cortex has better temporal resolution and is more sensitive to rapid temporal changes critical for speech processing, while the right hemisphere auditory cortex has higher spectral resolution and is more modulated by spectral changes, which optimize musical processing (Zatorre et al., 2002; Poeppel, 2003; Schönwiesner et al., 2005; Hyde et al., 2008). Thus, left auditory cortex has been found to be more responsive to phonemes than chords, while right auditory cortex is more responsive to chords than phonemes (Tervaniemi et al., 1999, 2000). This hemispheric specialization coincides with evidence from both auditory and visual domains, suggesting that the left hemisphere tends to be tuned to local features, while the right hemisphere is tuned to more global features (Sergent, 1982; Ivry and Robertson, 1998; Sanders and Poeppel, 2007).

Hemispheric differences in the present study for speech and music vary by location. We did not find any qualitative hemispheric differences between speech and music in the temporal lobe. Speech bilaterally activated lateral superior and middle temporal regions, while music bilaterally activated more dorsal medial superior temporal regions extending into the inferior parietal lobe. However, these bilateral findings should not be interpreted as evidence against hemispheric asymmetries for speech vs. music. The hemispheric differences widely reported in auditory cortex almost always are a matter of degree, e.g., phonemes and tones both activate bilateral superior temporal regions, but a direct comparison indicates a left hemisphere preference for the speech and a right hemisphere preference for the tones (Jäncke et al., 2002; Zatorre et al., 2002). These differences would not be reflected in our ALE results because both conditions reliably activate the same regions although to different degrees and the ALE method does not assign weight to coordinates (i.e., all the significant coordinates reported for contrasts of interest in the studies used) based on their beta or statistical values.

The frontal lobe results, however, did include some laterality differences of interest: passive listening to speech activated portions of the left inferior frontal gyrus (i.e., Broca's area), namely in the pars triangularis, significantly more than listening to music. A right inferior frontal gyrus cluster, extending into the insula, was activated significantly more for listening to music than speech. These findings in Broca's area coincide with Koelsch's neurocognitive model of music perception, in that right frontal regions are more responsive to musical stimuli and that the pars opercularis, but not the pars triangularis, is engaged in structure building of auditory stimuli (Koelsch, 2011). It is also noteworthy that the inclusion of a task altered hemispheric differences in the frontal lobes: the music discrimination tasks activated the left pars opercularis more than speech discrimination, while speech detection and memory tasks activated all of Broca's area (pars opercularis and pars triangularis) more than music detection and memory tasks; music detection and discrimination tasks, but not music memory tasks, activated the right inferior frontal gyrus more than corresponding speech tasks. These task-modulated asymmetries in Broca's area for music are particularly important when interpreting the rich electrophysiological literature of speech and music interactions. For example, both the early right anterior negativity (ERAN) and early left anterior negativity (ELAN) are modulated by speech and music, and are believed to have sources in both Broca's area and its right hemisphere homolog (Friederici et al., 2000; Maess et al., 2001; Koelsch and Friederici, 2003). Thus, the lateralization patterns found in the present study emphasize the need to consider that similar ERP effects for speech and music may arise from different underlying lateralization patterns that may be task-dependent.

### Speech vs. music in the anterior temporal lobe

Superior and middle posterior temporal regions on the banks of the superior temporal sulcus were preferentially activated in each speech condition compared to each corresponding music condition in the present meta-analysis. This is not surprising, as these posterior STS regions are widely implicated in lexical semantic processing (Price, 2010) and STS regions have been found to be more responsive to syllables than tones (Jäncke et al., 2002). Perhaps more interestingly, the bilateral anterior temporal lobe (ATL) also was activated more for each speech condition than by each corresponding music condition. The role of the ATL in speech processing is debated (e.g., Scott et al., 2000 cf. Hickok and Poeppel, 2004, 2007), but the ATL is reliably sensitive to syntactic structure in speech compared to several control conditions including word lists, scrambled sentences, spectrally rotated speech, environmental sounds sequences, and melodies (Mazoyer et al., 1993; Humphries et al., 2001, 2005, 2006; Xu et al., 2005; Spitsyna et al., 2006; Rogalsky and Hickok, 2009; Friederici et al., 2010; Rogalsky et al., 2011). One hypothesis is that the ATL is implicated in combinatorial semantic processing (Wong and Gallate, 2012; Wilson et al., 2014), although pseudoword sentences (i.e., sentences lacking meaningful content words) also activate the ATL (Humphries et al., 2006; Rogalsky et al., 2011). Several of the speech activation coordinates included in the present meta-analysis were from studies that used sentences and phrases as stimuli (with and without semantic content). It is likely that these coordinates are driving the ATL findings. Our finding that music did not activate the ATL supports the idea that the ATL is not responsive to hierarchical structure *per se* but rather needs linguistic and/or semantic information for it to be recruited.

### Speech vs. music in broca's area

There is no consensus regarding the role of Broca's area in receptive speech processes (e.g., Fedorenko and Kanwisher, 2011; Hickok and Rogalsky, 2011; Rogalsky and Hickok, 2011). Results from the present meta-analysis indicate that listening to speech activated both the pars opercularis and pars triangularis portions of Broca's area, while listening to music only activated the pars opercularis. The pars triangularis has been proposed to be involved in semantic integration (Hagoort, 2005) as well as in cognitive control processes such as conflict resolution (Novick et al., 2005; Rogalsky and Hickok, 2011). It is likely that the speech stimuli contain more semantic content than the music stimuli, and thus semantic integration processes may account for the speech-only response in pars triangularis. However, there was no significant difference in activations in the pars triangularis for the music discrimination and music detection tasks vs. passive listening to speech, and the music memory tasks activated portions of the pars triangularis more than listening to speech. These music task-related activations in the pars triangularis may reflect the use of semantic resources for categorization or verbalization strategies to complete the music tasks, but may also reflect increased cognitive control processes to support reanalysis of the stimuli to complete the tasks. The activation of the left pars opercularis for both speech and music replicates numerous individual studies implicating the pars opercularis in both speech and musical syntactic processing (e.g., Koelsch and Siebel, 2005; Rogalsky and Hickok, 2011) as well as in a variety of auditory working memory paradigms (e.g., Koelsch and Siebel, 2005; Buchsbaum et al., 2011).

### Implications for neuroanatomical models of speech and music

It is particularly important to consider task-related effects when evaluating neuroanatomical models of the interactions between speech and music. It has been proposed that inferior frontal cortex (including Broca's area) is the substrate for shared speech-music executive function resources, such as working memory and/or cognitive control (Patel, 2003; Slevc, 2012; Slevc and Okada, 2015) as well as auditory processes such as structure analysis, repair, working memory and motor encoding (Koelsch and Siebel, 2005; Koelsch, 2011). Of particular importance here is Slevc and Okada's (2015) proposal that cognitive control may be one of the shared cognitive resources for linguistic and musical processing when reanalysis and conflict resolution is necessary. Different tasks likely recruit cognitive control resources to different degrees, and thus may explain task-related differences for the frontal lobe's response to speech and music. There is ample evidence to support Slevc and Okada's hypothesis: classic cognitive control paradigms such as the Stroop task (Stroop, 1935; MacLeod, 1991) elicit overlapping activations in Broca's area when processing noncanonical sentence structures (January et al., 2009). Unexpected harmonic and melodic information in music interfere with Stroop task performance (Masataka and Perlovsky, 2013). The neural responses to syntactic and sentence-level semantic ambiguities in language also interact with responses to unexpected harmonics in music (Koelsch et al., 2005; Steinbeis and Koelsch, 2008b; Slevc et al., 2009; Perruchet and Poulin-Charronnat, 2013). The present results suggest that this interaction between language and music possibly via cognitive control mechanisms, localized to Broca's area, may be task driven and not inherent to the stimuli themselves. In addition, many language/music interaction studies use a reading language task with simultaneous auditory music stimuli; it is possible that a word-by-word presentation reading paradigm engages additional reanalysis mechanisms that may dissociate from resources used in auditory speech processing (Tillmann, 2012).

Slevc and Okada suggest that future studies should use tasks designed to drive activation of specific processes, presumably including reanalysis. However, the present findings suggest it is possible that these task-induced environments may actually drive overlap of neural resources for speech and music not because they are taxing shared sensory computations but rather because they are introducing additional processes that are not elicited during typical, naturalistic music listening. For example, consider the present findings in the left pars triangularis: this region is not activated during listening to music, but is activated while listening to speech. However, by presumably increasing the need for reanalysis mechanisms via discrimination or memory tasks, music does recruit this region.

There may be inferior frontal shared mechanisms that are stimulus driven while others are task driven: Broca's area is a diverse region in terms of its cytoarchitecture, connectivity and response properties (Amunts et al., 1999; Anwander et al., 2007; Rogalsky and Hickok, 2011; Rogalsky et al., in press). It is possible that some networks are task driven and some are stimulus driven. The hypotheses of Koelsch et al. are largely grounded in behavioral and electrophysiology studies that indicate an interaction between melodic and syntactic information (e.g., Koelsch et al., 2005; Fedorenko et al., 2009; Hoch et al., 2011). It is not known if these interactions are stimulus driven; a variety of tasks have been used in this literature, including discrimination, anomaly/error detection, (Koelsch et al., 2005; Carrus et al., 2013), grammatical acceptability (Patel et al., 1998a; Patel, 2008), final-word lexical decision (Hoch et al., 2011), and memory/comprehension tasks (Fedorenko et al., 2009, 2011). In addition, there is substantial variability across individual subjects, both functionally and anatomically, within Broca's area (Amunts et al., 1999; Schönwiesner et al., 2007; Rogalsky et al., in press). Thus, future within-subject studies are needed to better understand the role of cognitive control and other domain-general resources in musical processing independent of task.

Different tasks, regardless of the nature of the stimuli, may require different attentional resources (Shallice, 2003). Thus, it is possible that the inferior frontal differences between the music tasks and passive listening to music and speech are due to basic attentional differences, not the particular task *per se*. However, we find classic domain-general attention systems in the anterior cingulate and medial frontal cortex to be significantly activated across all conditions: music tasks, speech tasks, passive listening to music and passive listening to speech. These findings support Slevc and Okada's (2015) claim that domain-general attention mechanisms facilitated by anterior cingulate and medial frontal cortex are consistently engaged for music as they are for speech. Each of our music task conditions do activate these regions significantly more than the passive listening, suggesting that the midline domain-general attention mechanisms engaged by music can be further activated by explicit tasks.

### Limitations and future directions

One issue in interpreting our results may be the proximity of distinct networks for speech and music (Peretz, 2006; Koelsch, 2011). Overlap in fMRI findings, particularly in a meta-analysis, does not necessarily mean that speech and music share resources in those locations. It is certainly possible that the spatial resolution of fMRI is not sufficient to visualize separation occurring at a smaller scale (Peretz and Zatorre, 2005; Patel, 2012). However, our findings of spatially distinct regions for music and speech clearly suggest the recruitment of distinct brain networks for speech and music.

Another potential issue related to the limitations of fMRI is that of sensitivity. Continuous fMRI scanning protocols (i.e., stimuli are presented simultaneously with the noise of scanning) and sparse temporal sampling fMRI protocols (i.e., stimuli are presented during silent periods between volume acquisitions) are both included in the present meta-analyses. It has been suggested that the loud scanner noise may reduce sensitivity to detecting hemodynamic response to stimuli, particularly complex auditory stimuli such as speech and music (Peelle et al., 2010; Elmer et al., 2012). Thus, it is possible that effects only detected by a sparse or continuous paradigm are not represented in our ALE results. However, a comparison of continuous vs. sparse fMRI sequences found no significant differences in speech activations in the frontal lobe between the pulse sequences (Peelle et al., 2010).

Priming paradigms measuring neurophysiological responses (ERP, fMRI, etc.) are one way to possibly circumvent task-related confounds in understanding the neurobiology of music in relation to that of speech. Tillmann (2012) suggests that priming paradigms may provide more insight into an individual's implicit musical knowledge than is demonstrated by performance on an explicit, overt task (e.g., Schellenberg et al., 2005; Tillmann et al., 2007). In fact, there are ERP studies that indicate that musical chords can prime processing of target words if the prime and target are semantically (i.e., emotionally) similar (Koelsch et al., 2004; Steinbeis and Koelsch, 2008a). However, most ERP priming studies investigating music or music/speech interactions have included an explicit task (e.g., Schellenberg et al., 2005; Tillmann et al., 2007; Steinbeis and Koelsch, 2008a). It is not known how the presence of an explicit task may affect priming mechanisms via top-down mechanisms. Priming is not explored in the present meta-analysis; to our knowledge there is only one fMRI priming study of music and speech, which focused on semantic (i.e., emotion) relatedness (Steinbeis and Koelsch, 2008a).

The present meta-analysis examines networks primarily in the cerebrum. Even though almost all of the studies included in our analyses focused on cortical structures, we still identified some subcortical task-related activations: music detection compared to music passive listening activated the basal ganglia and music memory tasks activated the thalamus, hippocampus and basal ganglia compared to music passive listening. No significant differences between passive listening to speech and music were found in subcortical structures. These findings (and null results) in subcortical regions should be interpreted cautiously: given the relatively small size of these structures, activations in these areas are particularly vulnerable to spatial smoothing filters and group averaging (Raichle et al., 1991; White et al., 2001). There is also strong evidence that music and speech share subcortical resources in the brainstem (Patel, 2011), which are not addressed by the present study. For example, periodicity is a critical aspect of both speech and music and known to modulate networks between the cochlea and inferior colliculus of the brainstem (Cariani and Delgutte, 1996; Patel, 2011). Further research is needed to better understand where speech and music processing networks diverge downstream from these shared early components.

## Conclusion

Listening to music and listening to speech engage distinct temporo-parietal cortical networks but share some inferior and medial frontal resources (at least at the resolution of fMRI). However, the recruitment of inferior frontal speech-processing regions for music is modulated by task. The present findings highlight the need to consider how task effects may be interacting with conclusions regarding the neurobiology of speech and music.

### Conflict of interest statement

The authors declare that the research was conducted in the absence of any commercial or financial relationships that could be construed as a potential conflict of interest.

## Footnotes

^1^The music categories included studies with stimuli of the following types: instrumental unfamiliar and familiar melodies, tone sequences and individual tones. In comparison, the speech categories described below included studies with stimuli such as individual phonemes, vowels, syllables, words, pseudowords, sentences, and pseudoword sentences. For the purposes of the present study, we have generated two distinct groups of stimuli to compare. However, music and speech are often conceptualized as being two ends of continuum with substantial gray area between the two extremes (Koelsch, 2011). For example, naturally spoken sentences contain rhythmic and pitch-related prosodic features and a familiar melody likely automatically elicits a mental representation of the song's lyrics.
